# Response-adaptive randomization in clinical trials

**DOI:** 10.1177/09622802261486806

**Published:** 2026-09-29

**Authors:** David S. Robertson, Oleksandr Sverdlov, Sofía S. Villar, Hongjian Zhu

**Affiliations:** 1 MRC Biostatistics Unit, 47959 University of Cambridge, Cambridge, UK; 2 Advanced Quantitative Sciences, 33412 Novartis Pharmaceuticals Corporation, East Hanover, NJ, USA; 3 Statistics and Data Science, SystImmune Inc., Redmond, WA, USA

**Keywords:** Adaptive design, clinical trials, response-adaptive randomization, editorial, special collection

**Editorial note:** This special collection on response-adaptive randomization is made up of 19 articles spread over three issues: the special issue Volume 34, Number 9 (September 2025; 11 articles), one article in Volume 34, Number 10 (October 2025; Giovagnoli and Lupparelli), and this special issue (7 articles plus the editorial).

The traditional paradigm of equal, fixed randomization in clinical trials has long been considered the gold standard for ensuring internal validity and minimizing bias. However, the rise of precision medicine and a growing emphasis on the ethical imperative to protect trial participants have catalyzed a shift toward more flexible, data-driven approaches. Response-adaptive randomization (RAR) stands at the forefront of this evolution. By utilizing accumulating data within a trial to modify the probability of treatment assignment, RAR aims to achieve a dual objective: maintaining scientific rigor while skewing allocation toward treatments that (for example) appear to be more beneficial.

The genesis of this special collection can be traced back to a period of reflection within the statistical community. In 2023, Robertson et al.^1^ published a comprehensive review on RAR in Statistical Science, which served as a catalyst for renewed debate on the method's utility and limitations. This review was subsequently presented and discussed at a dedicated workshop on 29 February 2024 held in Cambridge, UK. It was in the interval between these two milestones that the idea for this special collection was conceived. Our hope was to issue a formal call for research that directly addressed the specific gaps and open areas highlighted during those discussions—moving beyond the well-trodden debates of the past to focus on the practical, methodological, and ethical work still required for the field to progress.

Despite the intuitive appeal of “learning while doing,” the implementation of RAR is also known to be not without controversy. Critics often point to potential tradeoffs, such as a loss of statistical power, the introduction of bias due to temporal trends, and the complexities of post-trial inference.^2^ In recent years, the dialogue has started to move beyond whether RAR should be used, and more toward how and when it can be implemented rationally, ethically, and robustly.

This special collection features three review articles and 16 original research articles and was curated to bridge the gap between theoretical statistical innovation and practical clinical application, offering a snapshot of the current state of the art while identifying the milestones required for future regulatory and clinical acceptance.

We called for the submission of:

Review papers describing the state of the art and open challenges: articles describing contemporaneous applications of RAR in clinical trials, best practice, existing software, etc.

Original research articles expanding the state of the art on RAR: New statistical methodology and their application to clinical trials including covariate-adjusted response adaptive methods, methods for handling of missing data for the implementation of RAR design, estimation and hypothesis testing issues after RAR (for example, the use of estimands as well as efficiency and validity in finite samples), guidance for simulation studies, use of machine learning or AI in RAR, and approaches to benchmark or compare RAR with other adaptive designs. Both illustrative application via simulation and direct application of new methodology to a specific clinical trial were welcome.

Three papers in the special collection provide systematic reviews of statistical methodology, implementation, and real-world applications of RAR in modern clinical research.

Mei et al.^3^ investigate the translation of RAR methodologies into practice. Spanning a decade from 2015 to 2024, this review identifies 19 trials that used RAR to allocate more patients to the superior treatment group. The authors provide a summary of various RAR design features, including a suite of under-explored challenges such as handling different types of outcomes, incorporating repeated measurements, and handling missing data issues in RAR trials. They also offer potential solutions to the identified challenges that can help clinical investigators implement the chosen RAR methodologies in practice.

Sverdlov et al.^4^ provide an empirical baseline regarding the clinical uptake and regulatory acceptance of RAR. By utilizing the Cortellis Regulatory Intelligence database, the authors identify 39 completed and ongoing RAR trials, examining their distribution across clinical phases and therapeutic areas. The added value of this study is its focus on regulatory impact. It documents, among others, instances where RAR designs provided pivotal evidence necessary for the approval of successful compounds, such as lemborexant for the treatment of insomnia, and lecanemab for early Alzheimer's disease.

Wilson et al.^5^ provide a comprehensive review of 65 RAR trials, highlighting the gap between planned RAR designs and their implementation and reporting. This paper quantifies the tangible benefits of RAR, such as a 22% reduction in the required sample size. At the same time, the authors provide a cautionary note regarding transparency in reporting—71% of RAR trials lacked clear statistical implementation details, which speaks to the importance of adopting more rigorous reporting standards for RAR, such as the Adaptive Designs CONSORT Extension.^6^

Current advancements in clinical trial methodologies focus on the integration of covariates to enhance both statistical efficiency and ethical rigor. The following research articles in the special collection reflect the paradigm shift toward precision medicine and optimal statistical efficiency in complex clinical trials. To advance precision medicine via continuous biomarker evaluation, Baldi Antognini et al.^7^ propose the biomarker-adjusted response-adaptive procedure, which sequentially minimizes the Euclidean distance to optimal allocations to estimate the optimal relative risk-based cutoff of a continuous predictive biomarker in binary response trials. Addressing the analytical challenges of high-dimensional patient profiles, Guo et al.^8^ introduce an innovative adaptive randomization approach with simultaneous covariate selection, effectively mitigating covariate over-incorporation and accelerating the convergence rate of imbalance measures via regularization. Further enhancing post-allocation inference, Tu and Ma^9^ explore robust, nonlinear machine learning-based covariate adjustments under the doubly-adaptive biased coin design (DBCD), employing sample-splitting techniques to alleviate stringent convergence requirements and achieve asymptotic normality. Extending adaptive frameworks to time-to-event endpoints, Mukherjee et al.^10^ develop CARA designs for semiparametric survival models, employing covariate-adjusted efficient response adaptive randomization design (ERADE) and DBCD rules to target optimal allocations under a Cox proportional hazards framework, thereby obviating restrictive parametric survival prerequisites. From a Bayesian perspective, Pei et al.^11^ refine CARA by strictly distinguishing predictive covariates—used to ethically skew allocation toward superior treatments—from prognostic covariates, which are strictly balanced to protect against confounding. To accommodate heteroscedasticity and improve the detection of treatment effect heterogeneity, Liu^12^ proposes the model-based Neyman allocation, a procedure derived to dynamically maximize the power of treatment–covariate interaction tests. Finally, bridging the gap between adaptive allocation and robust semiparametric inference, Xin and Ma^13^ provide a definitive proof that a stratified difference-in-means estimator, coupled with a stratified DBCD, achieves Armstrong's asymptotic efficiency bound under constrained optimization. Collectively, these contributions provide a rigorous theoretical foundation and versatile practical toolkits for designing highly efficient, covariate-adjusted, and ethical adaptive trials.

Two papers in the special collection focus on response-adaptive procedures for trial settings that are seeing recent increased popularity in practice: multi-arm multi-stage (MAMS) designs and master protocols. Liu and Coad^14^ consider the MAMS setting and extend Fisher's least significant difference method, allowing for the targeting of optimal allocations using RAR while still controlling the familywise error rate. Jacko et al.^15^ consider the master protocol setting where each subtrial is a single-arm study and show how the predictive probability of current study success can be used to define response-adaptive procedures.

Meanwhile, Giovagnoli and Lupparelli^16^ also consider using predictive probabilities (from a Bayesian viewpoint) to make RAR based on past data less myopic. Continuing further in making RAR non-myopic, Yi and Wang^17^ investigate optimal allocation using Markov decision processes and Thompson sampling, aiming to maximize average rewards while preventing very unbalanced group sizes. Finally, an open question regarding targeting optimal allocations is how to achieve the Cramér-Rao lower bound on the (asymptotic) variance of the allocation performance for multi-arm trials—in the two-arm setting this was answered with the ERADE proposed by Hu et al.^18^ in 2009. Alkhnefr et al.^19^ derive the generalization of ERADE to the multi-arm setting and demonstrate its effectiveness in finite sample settings.

As the use of RAR becomes more common in practice, questions around practical implementation are coming more to the fore, as reflected in four papers in the special collection. Tang et al.^20^ tackle the question of how long the burn-in period (i.e. the equal randomization stage) should be when using Bayesian RAR. Meanwhile, Tackney and Villar^21^ explore how to account for missing response data when using RAR, focusing on the pros and cons of using imputation. Das et al.^22^ focus on rare-disease trials, where the small numbers of participants impose practical constraints on the achievable randomization ratios when using RAR. Finally, Zhu et al.^23^ revisit the historical (and continuing) controversy surrounding the use of RAR in practice, as exemplified by Korn and Freidlin's 2011 article.^24^ An unpublished original response to that paper by Zhu et al. is presented for the first time in this special collection, accompanied by a present-day commentary that revisits this discussion 15 years later, exploring the nuances and complexities of implementing RAR in the context of contemporary clinical trial design.

The 19 papers featured in this special collection illustrate a vibrant and rapidly maturing field. From the rigorous empirical reviews that document the growing regulatory footprint of RAR to the cutting-edge methodologies that integrate machine learning and covariate adjustment, it is clear that response-adaptive designs are no longer merely theoretical curiosities. They are becoming practical tools in the quest for more efficient and patient-centric clinical research.

However, as several authors in this collection note, significant work remains. The practical hurdles of missing data and the historical debates regarding efficiency versus ethics—most notably revisited in this collection's historical note—remind us that methodological progress must be accompanied by clarity and transparency. The findings presented here emphasize that there is no “one-size-fits-all” design; instead, the choice of a randomization procedure must be a deliberate act, centered on the specific ethical and scientific needs of the trial at hand.

We hope that this collection serves as both a practical toolkit for trialists and a catalyst for further research. As we look toward the future, the integration of RAR into broader frameworks such as the International Council for Harmonisation E20 guidance on adaptive designs^25^ and the continued refinement of reporting standards will be critical. By surfacing these challenges and showcasing potential solutions, we believe this special collection provides a necessary foundation for the next generation of rational and ethical clinical trial designs.
